# Carvacrol alters soluble factors in HCT-116 and HT-29 cell lines

**DOI:** 10.3906/sag-1907-173

**Published:** 2020-02-13

**Authors:** Ahu PAKDEMİRLİ, Caner KARACA, Tolga SEVER, Ezgi DASKIN, Asım LEBLEBİCİ, Türkan YİĞİTBAŞI, Yasemin BAŞBINAR

**Affiliations:** 1 Department of First and Emergency Aid, Vocational School of Health Services, Dokuz Eylül University, İzmir Turkey; 2 Department of Basic Oncology, Institute of Health Sciences, Dokuz Eylül University, İzmir Turkey; 3 Department of Translational Oncology, Institute of Health Sciences, Dokuz Eylül University, İzmir Turkey; 4 Department of Biochemistry, Faculty of Medicine, İstanbul Medipol University, İstanbul Turkey; 5 Department of Translational Oncology, Institute of Oncology, Dokuz Eylül University, İzmir Turkey

**Keywords:** Carvacrol, phytochemical, colon cancer, soluble factors

## Abstract

**Background/aim:**

Natural products are popular insights for researchers to investigate promising anti-cancer agents since some of these substances have lesser adverse effects restricting the treatment than traditional chemotherapeutic agents. A well-known monoterpene Carvacrol, widely consumed in Mediterranean cuisine and lower risks of cancer, has efficient anticancer effects. However, the mechanism of action is yet to be discovered.

**Materials and methods:**

The investigation aims to illuminate a new perceptive in the role of this substance on colorectal cancer treatment, by the means of differences in a well-defined range of soluble factors. Carvacrol effect on both HT-29 and HCT-116 cell lines was evaluated on proliferation and the IC50 values were calculated by the RTCA xCELLigence device. Then MAGPIX assay was performed to obtain the changes in soluble factors of the cell lines.

**Results:**

The Multiplexing assay suggests some of these factors were altered in favor of surviving and proliferation in aggressive cell line HCT-116 whereas they were altered against these characters in HT-29, were correlated with the increased IC50 concentration of HCT-116 in carvacrol treatment.

**Conclusion:**

The current study indicates that differences in the levels of these soluble factors could modulate the anticancer effect related to carvacrol.

## 1. Introduction

Colon cancer has become a major public health issue with 1,849,518 new cases in 2018. 10.2% of all cancer deaths in the same year were caused by this malign disease which brings it to the second rank in cancer related deaths [1]. Several factors (i.e. age, familial cancer syndromes, inflammatory bowel disease, dietary factors, hereditary polyposis conditions) affect the development of this malignancy [2]. Nowadays, the most accepted therapies are surgery, radiotherapy, and chemotherapy such as 5-fluorouracil and oxaliplatin [3,4]. However, all these procedures have their own difficulties, the adverse effects related to systemic chemotherapy are much restrictive due to their mortality. Since some of the natural products as chemotherapeutic agents indicate lower side effects on patients, they are promising anticancer agents and safer sources of cancer treatments. Moreover, such phytochemicals are also related with chemopreventive agents because of their eﬀiciency and low toxicity. Therefore, the scientists focus on evaluating phytochemicals with multitargeted potentials for eﬀective chemoprevention [5].

Terpenes (C10H16) illustrated enormous effectiveness as chemopreventive agent in the last years however the mechanism of action is not clear yet (a subclass of this family, terpenoids, has been characterized as monoterpenes, diterpenes, oligoterpenes, and polyterpenes). They involve roughly 25,000 chemical structures which are used in pharmaceutical industries [6]. Carvacrol is also a member of this family. The substance presents in various sources that are extensively tested with enormous biological activities [7]. Mediterraneans regularly consume this product with oregano spice that presents a lesser risk of colorectal cancer compared to the other local cuisines [8]. Nowadays, it is well-known that the carvacrol in this spice is the one reason that the substance has preventive properties. It basically protects mammalian cells against DNA strand breaks along with rosmarinic acid [9,10]. Moreover, several studies point out that carvacrol illustrates further anticancer effects such as genotoxic, cytotoxic, and proapoptotic activity to cancer cells, in a dose-dependent manner. Additionally, it has also an important preventive role in cell invasion by down regulating of matrix metalloprotease 2 and 9 expressions [11].

Tumor microenvironments involve various cell types and extracellular matrix substances in the niche of cancer such as soluble factors. The soluble factors which could be related to cell proliferation are granulocyte-macrophage colony-stimulating factor (GM-CSF), leptin, prolactin and soluble vascular endothelial growth factor receptor-2 (sVEGFR2) evaluated [12]. Prolactin may involve in the genetic and molecular mechanisms for regulating the proliferation and growth of neoplastic and normal epithelial cells in the gastrointestinal tract [13]. Moreover, leptin is crucial for colorectal cancer (CRC) growth in obese patients [14]. On the other hand, sVEGFR2 involves the extracellular domains of the receptor but lacks the tyrosine kinase domain [15]. GM-CSF has an important role in hematopoiesis and immune modulation. GM-CSF also stimulates the progression of immune-independent tumors by inducing tumor growth, metastasis, and promoting tumor microenvironments. Hence several studies suggest that this factor triggers some effects on tumor progression [16].

Since these soluble factors manage the tumor microenvironment and the behavior of cancer as mentioned previously, the present study investigates the changes in these soluble factors when HT-29 and HCT-116 colorectal adenocarcinoma cell lines are treated with carvacrol in a dose-dependent manner. We suggest that the results could lead us one step further to discover the mechanism of action of carvacrol through the soluble factors.

## 2. Materials and methods

### 2.1. Cell culture

Human colorectal carcinoma, HCT-116 (ATCC CCL-247) and HT-29 (ATCC HTB-38) cell lines were purchased from American Type Culture Collection (ATCC) (Rockville, CT, USA). They were cultured in McCoy’s-5a modified medium (BiochromGbmH, Berlin, Germany) supplemented with 1% penicillin/streptomycin (BiochromGbmH, Berlin, Germany) and 10% fetal bovine serum (FBS, Cegrogen Biotech GmbH, Stadtallendorf, Germany). Cells were incubated at 37 °C in a humidified 5% CO2 incubator.

### 2.2. Cell viability

The antiproliferative effect of carvacrol (C7727, Sigma-Aldrich, St Louis, MO, USA) has been studied on HCT-116 and HT-29 colorectal adenocarcinoma cell lines. The cell lines were seeded in a triplicate in 96-well plate (10,000 cells per well). Cell viability was tested after 48 h of incubation with 25–200 µM carvacrol via xCELLigence real-time cell analysis instrument (ACEA Biosciences, Inc (ACEA), San Diego, California, USA).

### 2.3. Analysis of soluble factors via multiplexing assay

Colorectal adenocarcinoma cell lines, HCT-116, and HT-29 were treated with carvacrol at IC50 values individually. Both carvacrol treated and nontreated control groups were seeded into wells and run in triplicate. The fluorescent signal was measured by a CCD imager and the concentrations of the analytes were determined by MAGPIX xPONENT software. The results were normalized to total protein concentration estimated with Bradford assay.

### 2.4. Statistical analysis

SPSS version 24.0 (IBM Corp., Armonk, NY, USA) was used for all analyses. Willcoxon signed ranks nonparametric test was used to determine the significance between the application of different doses of carvacrol on different cell lines individually and its control group. The data in the Figure are represented as the mean ± standard error of the mean. In the study, all tests were performed in triplicate. The level of statistical significance was set at P < 0.05 .

## 3. Results

The current investigation draws attention to the carvacrol effect in proliferation and also points out its mechanism of action through the alterations in the level of soluble factors in colorectal cancer cell lines with different phenotypes. The methodology indicates the IC50 values for carvacrol and follows through the MAGPIX multiplexing assay to illuminate the changes in soluble factors in response to IC50 values of carvacrol on HCT-116 and HT-29 colorectal cancer cell lines.

### 3.1. The effect of carvacrol on cell proliferation of colorectal cancer cells

The in vitro proliferation activity doses of carvacrol on HCT-116 and HT-29 cell lines were measured by using the xCELLigence real-time cell analysis instrument. After 48 h of incubation with carvacrol, IC50 values were found as 92 µM for HCT-116 and 42 µM for HT-29 cell lines. The results are illustrated in Figure.

### 3.2. Carvacrol effect on soluble factors in colon cancer cell lines with different phenotypes

The soluble factors were analyzed to evaluate the cellular response leading to cytotoxicity in both cell lines and the difference between the response intensity to carvacrol. The measurements in samples were achieved by MAGPIX multiplexing assay. The Table which shows the normalized data illuminates that the secretion of several soluble factors related to growth and survival such as prolactin, leptin, and GM-CSF were increased in carvacrol treated HCT-116 while they were decreased in carvacrol treated HT-29 comparing to nontreated control groups. Prolactin, leptin, and GM-CSF concentrations were measured as 57.5, 9, and 82 pg/mL in HCT-116 and, 147, 17.74, and 104 pg/mL in HT-29 with no carvacrol concentration. These values raised in concentration to 117, 23, 105 pg/mL (P = 0.028 for all concentrations), in response to 92 µM and the IC50 value of carvacrol, respectively. However, they decreased to 53.49, 14.49, and 96 pg/mL concentrations (P = 0.028 for all concentrations) compared to the control concentrations in HT-29 treated with 42 µM of the phyto-compound. However, proliferative and survival factor TGF-α decreased in both cell lines, correlated with earlier proliferation assay. This factor was estimated as 67 pg/mL for HCT-116 and 106.2 pg/mL for HT-29. Both values diminished where the concentration for HCT-116 treated with 92 µM substance was 24 pg/mL (P = 0.028) and 26 pg/mL (P = 0.109) for HT-29 treated with 42 µM of carvacrol.

## 4. Discussion

The present study focused on the effect of carvacrol in proliferation and also illuminated its mechanism of action through the change in the level of soluble factors in colorectal cancer cell lines with different phenotypes. Both of these cell lines are treated with different doses of carvacrol which are 25 µM, 50 µM, 100 µM, 200 µM in xCELLigence real-time cell analysis instrument which measures the proliferation once per 15 min. The results signify that the proliferation levels of both cell lines decreased while the concentration level of carvacrol increased. A previous study points out that a carvacrol rich essential oil of origanumacutidens has also significant anti-proliferative effect against the HT-29 cell line. The most active concentration of this oil has been found as 100 μg/mL [17]. Moreover, similar to our results, Kai Fana et al. have studied the antiproliferative effect of carvacrol on HCT-116 and they detected the IC50 dose as 544.4 µM which is an even higher concentration comparing to our result [11]. Even though carvacrol has a significant antiproliferative effect on both of these cell lines in the dose-manner, our results indicate that it is more efficient against HT-29 since IC50 value of HCT-116 doubles the other line.

**Figure 1:**
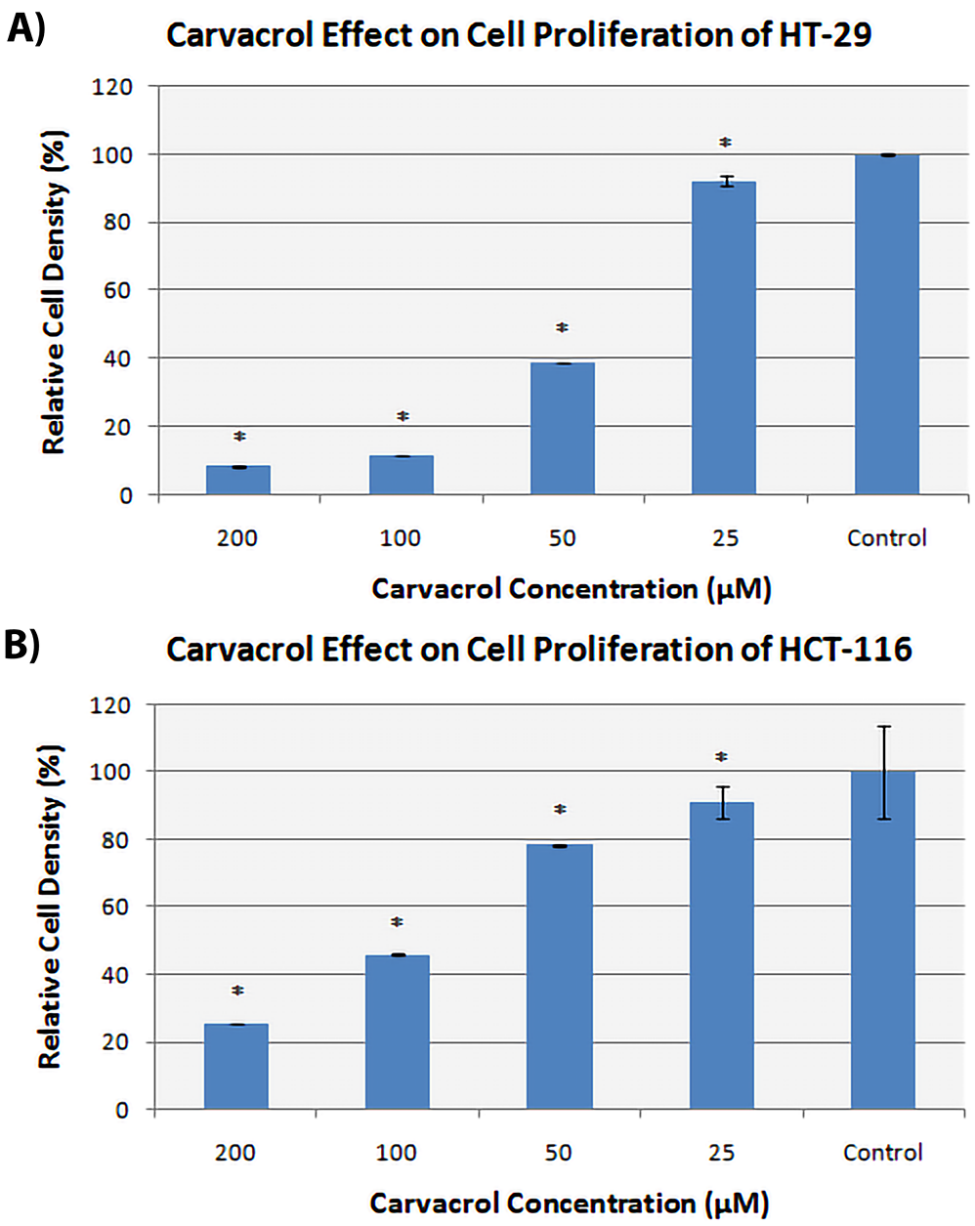
The antiproliferation effect of carvacrol in colorectal cancer. Dose-dependent cytotoxicity activity of carvacrol on HT-29 (A). The HCT-116 cell line was incubated with increasing concentrations of curcumin (B). After 48 h of incubation at 37 °C and 5% CO2 the cell viability was measured by xCELLigence real-time cell analysis instrument.

**Table 1:** Statistically significant concentration levels of soluble factors in carvacrol treated samples compared to nontreated controls. All measurements were achieved by MAGPIX multiplexing assay. Red indicates a decrease whereas green illustrates an increase in concentrations compared to controls.

Groups	Soluble factors	Control (pg/mL)	Treated (pg/mL)	P- value	Evaluation
HT-29	GMCSF	104.0000	96.0000	0.028	decreased	FGF basic	55.4967	41.2467	0.028	decreased	GCSF	177.9967	116.7200	0.028	decreased	HGF	35.7567	27.9967	0.028	decreased	Leptin	17.7467	14.4967	0.028	decreased	Osteopontin	49.5000	61.6200	0.028	increased	PECAM1	72.7467	77.9967	0.028	increased	Prolactin	147.0000	53.4967	0.028	decreased	SCF	29.7467	46.2467	0.028	increased	sEGFR	57.9967	41.4967	0.028	decreased	sHER2neu	35.2467	22.9967	0.028	decreased	sIL6Ra	36.9967	12.4967	0.028	decreased	sTIE2	45.2467	44.0000	0.028	decreased	sVEGFR1	77.9967	10.4967	0.028	decreased	sVEGFR2	30.4967	14.9967	0.028	decreased	IL18	30.0000	19.4967	0.028	decreased	TGF	106.2467	25.9967	0.109	decreased	VEGFC	16.9967	40.9967	0.002	increased
	HCT-116	GMCSF	82.0000	105.0000	0.028	increased	IL8	30.0000	9.0000	0.028	decreased	FGF basic	22.4967	47.9967	0.028	increased	GCSF	116.2467	117.4967	0.028	increased	HGF	12.5000	15.0000	0.028	increased	Leptin	8.9967	23.4967	0.028	increased	Osteopontin	40.9933	53.5000	0.028	increased	PECAM1	16.4967	27.9967	0.028	increased	Prolactin	57.4967	117.0000	0.028	increased	SCF	44.7467	61.9967	0.028	increased	sHER2neu	28.2467	78.2467	0.028	increased	sIL6Ra	23.9967	27.4967	0.028	increased	sTIE2	39.2467	34.0000	0.028	decreased	sVEGFR1	72.2467	66.4967	0.028	decreased	sVEGFR2	10.0000	21.9967	0.028	increased	TGF	66.9967	23.9967	0.028	decreased

The data of proliferation assay on the carvacrol effect point out that the phyto-compound has a significant role in both HCT-116 and HT-29 cell lines. However, the intensity of the cytotoxic responses is different since a more concentrated dose is required in HCT-116. Considering this result, the cellular response was evaluated by the means of secreted soluble factors via multiplexing assay (Table). The normalized data illuminate that the secretion of several soluble factors such as prolactin, leptin, and GM-CSF were increased in carvacrol treated HCT-116 while they were decreased in carvacrol treated HT-29 comparing to nontreated control groups. Considering this mitogenic effect, survival character was more abundant in HCT-116 whereas it reduced in HT-29 in response to the phyto-compound. Therefore, HCT-116 could be evaluated more resistant and aggressive in carvacrol treatment. Nevertheless, a significant decrease in proliferative and survival protein TGF-α and the increase in antigrowth factors VEGFR2 were correlated with the total cytotoxic effect in the final response.

The literature mostly confirms the results which we have obtained in the current study. The sVEGFR2 fragments, transfected to tumor cells, are secreted to the extracellular matrix and inhibit angiogenesis in vivo [18]. Another soluble factor increased in HCT-116, prolactin is actively involved in tumorigenesis in several cancers. The current research also points out that the prolactin level of HT-29 is dramatically lower compared to the control group. On the other hand, the level of this factor is enormously enhanced in the HCT-116 cell line. Moreover, the scientists investigated in improvement cures to control tumor growth via reducing the prolactin production [19]. The data introduced in the current study illuminates the levels of prolactin increased in HCT-116 as a result of favoring survival and proliferation, whereas it was decreased in HT-29. Therefore this growth-favored factor indicates that it is possible to enhance carvacrol treatment. In addition, the expression level of leptin was measured in lower levels in the HT-29 cell line compared to its control group. However, HCT-116 has an elevated level of this soluble factor. Since, previous reports suggest that leptin is overexpressed in various types of cancer cells and plays a role in the development and progression of a variety of malignancies including colon cancer. Our current study also reveals another reason why HCT-116 cell line was more resilient than HT-29 against this compound [20,21]. GM-CSF was another growth factor that decreased in HT-29 whereas it increased in HCT-116. In addition to the earlier studies indicating that neutralizing GM-CSF reduced the proliferation, angiogenesis, and colonic epithelial cells (CECs) in neoplasia, our data point out that this factor is also an accessory after the fact along with leptin and prolactin [22].

In conclusion, different colorectal cancer cell lines respond in distinct ways to this phytochemical compound. On one hand, the antiproliferative feature of carvacrol has been shown in both cell lines, on the other hand the HCT-116 response is more resistant to carvacrol treatment compared to HT-29 cell line. On the basis of present data, our results revealed that the alteration in levels of soluble factors in the HCT-116 cell line enhances the ability to proliferate and survive. Even though some of these factors slightly favored to tumor growth for HCT-116, carvacrol affects both cell lines as an antiproliferative agent. Therefore this study also suggests that some soluble factors could be drug targets to enhance the effectiveness of carvacrol. 

## Acknowledgment

We sincerely thank Gizem Çalıbaşı Koçal, Tuğba Uysal, Mahdi Akbarpour, and Ece Çakıroğlu for their contribution in laboratory work.

## References

[ref0] (2018). GLOBOCAN estimates of incidence and mortality worldwide for 36 cancers in 185 countries. Global cancer statistics.

[ref1] (2015). Sikka S. Epidemiology of colorectal cancer — incidence, lifetime risk factors statistics, and temporal trends.

[ref2] (2014). Colorectal cancer-review. Laeknabladid.

[ref3] (2015). Identification of anti-metastatic drug and natural compound targets in isogenic colorectal cancer cells. Journal of Proteomics.

[ref4] (2012). Natural products for cancer prevention. Seminars in Oncology Nursing.

[ref5] (2011). Use of terpenoids as natural flavouring compounds in food industry. Recent Patents on Food, Nutrition & Agriculture.

[ref6] (2012). Terpenoids: natural products for cancer therapy. Expert Opinion on Investigational Drugs.

[ref7] (1989). Mediterranean diet and cancer. European Journal of Clinical Nutrition.

[ref8] (2007). DNA-protective effects of two components of essential plant oils carvacrol and thymol on mammalian cells cultured in vitro. Neoplasma.

[ref9] (2007). The interference of rosmarinic acid in the DNA fragmentation induced by osmotic shock. Frontiers in Bioscience.

[ref10] (2015). Carvacrol inhibits proliferation and induces apoptosis in human colon cancer cells. Anticancer Drugs.

[ref11] (2019). New insight of tumor microenvironment in non-small cell lung cancer. Journal of Basic and Clinical Health Sciences.

[ref12] (1995). Expression of prolactin and growth hormone receptor genes and their isoforms in the gastrointestinal tract. American Journal of Physiology.

[ref13] (2015). Obesity-related colorectal cancer: The role of leptin. Annals of Coloproctology.

[ref14] (2013). Prognostic and predictive value of VEGF, sVEGFR-2 and CEA in mCRC studies comparing cediranib, bevacizumab and chemotherapy. British Journal of Cancer.

[ref15] (2016). Stimulatory versus suppressive effects of GM-CSF on tumor progression in multiple cancer types. Experimental and Molecular Medicine.

[ref16] (2017). Real-time cell analysis of the cytotoxicity of origanumacutidens essential oil on HT-29 and HeLa cell lines. Turkish Journal Of Pharmaceutical Sciences.

[ref17] (2004). In vivo inhibition of tumor angiogenesis by a soluble VEGFR-2 fragment. Experimental and Molecular Pathology.

[ref18] (2015). A novel multiplexed immunoassay identifies CEA, IL-8 and prolactin as prospective markers for Dukes’ stages A-D colorectal cancers. Clinical Proteomics.

[ref19] (2007). Leptin acts as a mitogenic and antiapoptotic factor for colonic cancer cells. British Journal of Surgery.

[ref20] (2007). The anti.apoptotic and growth stimulatory actions of leptin in human colon cancer cells involves activation of JNK mitogen activated protein kinase, JAK2 and PI3 kinase/Akt. International Journal of Colorectal Disease.

